# Label-Free Quantitative Proteomic Analysis of *Puccinia psidii* Uredospores Reveals Differences of Fungal Populations Infecting Eucalyptus and Guava

**DOI:** 10.1371/journal.pone.0145343

**Published:** 2016-01-05

**Authors:** Maria Carolina Quecine, Thiago Falda Leite, Andressa Peres Bini, Thais Regiani, Lívia Maria Franceschini, Ilara Gabriela Frasson Budzinski, Felipe Garbelini Marques, Mônica Teresa Veneziano Labate, Simone Guidetti-Gonzalez, David Henry Moon, Carlos Alberto Labate

**Affiliations:** Departament of Genetics, Escola Superior de Agricultura “Luiz de Queiroz”, Universidade de São Paulo, Piracicaba -SP, Brazil; Institute for Sustainable Plant Protection, C.N.R., ITALY

## Abstract

*Puccinia psidii* sensu lato (s.l.) is the causal agent of eucalyptus and guava rust, but it also attacks a wide range of plant species from the myrtle family, resulting in a significant genetic and physiological variability among populations accessed from different hosts. The uredospores are crucial to *P*. *psidii* dissemination in the field. Although they are important for the fungal pathogenesis, their molecular characterization has been poorly studied. In this work, we report the first in-depth proteomic analysis of *P*. *psidii* s.l. uredospores from two contrasting populations: guava fruits (PpGuava) and eucalyptus leaves (PpEucalyptus). NanoUPLC-MS^E^ was used to generate peptide spectra that were matched to the UniProt *Puccinia* genera sequences (UniProt database) resulting in the first proteomic analysis of the phytopathogenic fungus *P*. *psidii*. Three hundred and fourty proteins were detected and quantified using Label free proteomics. A significant number of unique proteins were found for each sample, others were significantly more or less abundant, according to the fungal populations. In PpGuava population, many proteins correlated with fungal virulence, such as malate dehydrogenase, proteossomes subunits, enolases and others were increased. On the other hand, PpEucalyptus proteins involved in biogenesis, protein folding and translocation were increased, supporting the physiological variability of the fungal populations according to their protein reservoirs and specific host interaction strategies.

## Introduction

*Puccinia psidii* sensu lato (s.l.) is a biotrophic rust fungi native to South America, where it was first described in guava [1], causing severe infections resulting in losses up to100% of the fruits [2]. Eucalyptus rust is apparently a specialization of *P*. *psidii* genotype evolved from rust occurring in Myrtaceae in South America, enabling it to ‘host jump’ and infect *Eucalyptus*, an introduced Myrtaceae species to Brazil at the end of the 19^th^ century [3, 4]. Likely, some isolates from guava did not infect eucalyptus and vice versa [4] indicating an occurrence in the evolution of a host-specific genotype. Since then, rust has become one of the most important eucalyptus diseases, principally attacking nurseries and young plants, decreasing productivity, and in some cases, causing the death of the highly susceptible plants [5].Guava and other Myrtaceae from South America most likely served as the sources of inoculum during the adaptation of *P*. *psidii* to eucalyptus [6].

Recently, the genetic variability in *P*. *psidii* s.l. populations infecting different host plants was evaluated and proved that, in general, populations collected from guava had a greater level of diversity than populations from Eucalyptus *spp*. [6]. The classification of this fungus has been deeply debated. Morphological and molecular studies indicate that *Puccinia* is polyphyletic group [7, 8, 9], named *P*. *psidii* s.l. complex that is able to infect a great range of hosts from the Mytaceaes family. Thus, the disease caused by the fungus currently treated as *P*. *psidii* s.l. is best referred to as Myrtle rust [10].

*P*. *psidii* s.l. is considered an autoecious species with an incomplete life cycle (the spermogonia stage may be absent).The aecial state is extremely rare [5].The uredia are the most common and principal *P*. *psidii* s.l. dissemination structure and are constantly produced during natural and artificial inoculations [4]. Moreover, the uredosporic pustules are the only visual rust symptom that can be used to diagnose infection under field conditions [5, 11]. The uredospore protein profile may play a key role in the establishment of the disease, because they are the first fungal structures that have contact with the host before infection starts, and may carry some key proteins with respect to the host, because the rust uredopores germination is strongly dependent on physical and chemical signals from the host [12].

The physiological variability of *P*. *psidii* s.l.uredospores according to the host has been described previously [5, 13, 14]. Coelho et al. [13] identified three groups of rust biotypes, each pathogenic on different host combinations: *Psidium guajava* only, *Eucalyptus grandis* and *P*. *guajava* or *E*. *grandis* and *Sizygium jambos*. Despite the importance of the uredospore as the primary source of fungal inoculum, little is known about the molecular features of *P*. *psidii* s.l. uredospores or how they relate to its many host organisms, for instance guava and eucalyptus.

Global proteomic analysis can provide the framework for an in-depth understanding of the cellular processes and organization of a particular organism. The complexity of the proteome exceeds that of the genome when protein isoforms, alternative splicing variants, and post-translational modifications are taken into account [15]. However, proteomic analysis of filamentous fungi is still lacking in this field [16]. Among the filamentous fungi, the proteomes of the Ascomycota have been studied more intensively than those of Basidiomycota, most likely due to the number of pathogenic fungi that are found in Ascomycota [17]. Gradually, the proteomes of filamentous fungi are being revealed, including the mycelia proteome [18, 19, 20], secretomes [20, 21] and sub-proteomes [22] of a wide range of species, such as *Aspergillus* spp. [23, 24, 25, 26], *Botrytis cinerea* [17], *Neurospora crassa* [22], *Penicillium expansum* [27], *Pleurotus sapidus* [28], *Sclerotinia sclerotiorum* [20], *Terebralia palustris* [29], *P*. *triticina* [30]**,** *Blumeria graminis* [31], *Verticillium albo-atrum* [32], and others.

In the present work, we report the first label free proteomic quantification of *P*. *psidii* s.l. uredospores from two contrasting fungal populations with known physiological variability that reflects specific molecular interactions directed by the host. The results obtained allowed the identification of pathogenicity or virulence factors of this important pathogen and their correlation with the fungal physiological variation as a possible specific host interaction strategy during the infection process.

## Materials and Methods

### Pathogen material

*P*. *psidii* uredospores were collected from *E*. *grandis* leaves (PpEucalyptus) in the experimental area belonging to FuturaGene, Itapetininga-SP, Brazil in accordance with Dr. Esteban R. Gonzalez (FuturaGene Corporation Manager). Uredospores from *P*. *guajava* (PpGuava) where isolated from fruits of trees located in the Campus of ESALQ/USP, Piracicaba-SP, Brazil in accordance with the Chief of the Intitution Dr. José Caixeta Filho. Both fields do not involve endangered or protected species. Infected leaves and fruits from *E*. *grandis* and *P*. *guajava* tree`s were individually collected. We sampled three plants per species, a bulk of leaves or fruits from each plant considered as an independent biological replicate, totalizing three biological replicates per population sample. The uredospores samples from each replicate were divided, and half was immediately frozen at -80°C for proteomic analysis. The remaining sample was dehydrated in silica gel for 48 h at 4°C and frozen at -80°C for light microscopy analysis and for infection assay in *E*. *grandis*.

### Light microscopy analysis

The morphology of the PpEucalyptus and PpGuava uredospores and the germination viability assays were performed in water-agar medium (8 g/L). The uredospores from each population were suspended in mineral oil (Invitrogen) and 400 μL of each suspension (10^4^ uredospores/mL), spread onto the medium and incubated at 22°C in the dark for 24 h. The uredospores were analyzed using a light microscope (Axiophot II; Zeiss). For morphological analysis, we measured the length and width of 50 uredospores from each population, and the viability of the uredospores was confirmed by the presence of germ tubes after 24 h.

### Plant-infection assay

Eighteen highly susceptible *E*. *grandis* plantlets (monoprogenie D901) were grown under greenhouse conditions for 120 days and were then transferred to a controlled growth chamber (E15, Conviron),where they were maintained under a 12 h photoperiod (200 μmol m^-1^ s^-1^) at 20°C for acclimatization. After seven days, the PpEucalyptus and PpGuava uredospore suspensions containing 10^4^ uredospores mL^-1^ and 0.05% Tween 20 were sprayed onto six plantlets, two plants per biological sample. To maintain a high level of humidity, the plants were enclosed in transparent plastic bags for the first 48 h, with the first 24 h occurring in complete darkness at 20°C. Thereafter, the plants were returned to the previously described growth conditions. The control plantlets were treated in the same way, but were sprayed only with the 0.05% Tween solution. The evolution of the infection was monitored every two days for two weeks.

### Protein extraction and trypsin digestion

Proteins were extracted from uredospores according to the protocol described by Damerval et al. [33] with some modifications. Uredospore samples (1 g) were ground into a powder in liquid nitrogen and homogenized in 30 mL of TCA solution (10% (w/v) trichloroacetic acid and 0.07% (v/v) 2-mercaptoethanol in acetone) per biological replicate. The samples were then incubated for 1 h at -20°C, followed by centrifugation for 20 min at 16,000 *g* at 2°C. The supernatant was discarded, and the pellet was suspended in 30 mL of 100% cold acetone containing 0.07% (v/v) 2-mercaptoethanol. The pellets were incubated at -20°C, for one h, followed by centrifugation for 20 min at 16,000 *g* at 2°C. The pellet was again suspended in 30 mL of 100% cold methanol and incubated for one hour at -80°C. After centrifugation at 16,000 *g* for 20 min at 2°C, the supernatant was discarded, and the resulting pellet was lyophilized.

The dried pellets were solubilized in 800 μL of TCT buffer [7 M urea, 2 M thiourea, 10 mM DTT and 0.4% (v/v) Triton X-100]. Complete protein solubilization was achieved by vigorous shaking using a vortex. Protein extracts were desalinized using Amicon Ultra-0.5 mL 3K-NMWL filter devices (Millipore Corporation).

The total protein concentration was determined using an Agilent Bioanalyzer 2100 (Agilent) and the 230 kDa protein kit. Fifty micrograms of protein was denatured with 25 μL of 0.2% RapiGest (RapiGest SF, Waters), reduced with 2.5 μL of 100 mM dithiothreitol and alkylated with 2.5 μL of 300 mM iodoacetamide. Trypsin digestion was performed with sequencing Grade Modified Trypsin (Promega) at a 1:100 (w/w) enzyme: protein ratio. After digestion, 10 μL of 5% (v/v) trifluoroacetic acid was added to the digested mixture to hydrolyze the RapiGest. The peptide mixture was then desalted using PepClean C 18-columns (Thermo-Fisher Scientific). The final volume of 50 μL was obtained by the addition of 20 mM ammonium formate (pH 10) solution containing 1 pM rabbit phosphorylase (internal standard to data normalization and label free protein quantification- P00489) to the lyophilized, desalted peptide sample.

### MS^E^ analysis

Mass spectra of the peptide fragments were acquired by reverse-phase ultraperformance liquid chromatography (2D Technology nanoACQUITY-Waters). First dimension separation was achieved in an XBridge BEH130 C18 5μm 300 μm x 50 mm column. Elution was performed using 5 different binary gradients with 20 mM pH 10 ammonium formate in acetonitrile, at a flow rate of 2 μL/min. Eluted peptides from the first dimension column were trapped in a Symmetry C18 5 μm 180 μm x 20 mm column and diluted, online, with acetonitrile containing 0.1% formic acid. Second dimension separation was performed in a HSS T3 1.8 μm 75 μm x 100 mm column, using a binary gradient from 3 to 85% of acetonitrile with 0.1% formic acid, during 52 min, at a flow rate of 350 μL/min.

Mass spectrometry acquisition was achieved in a Synapt G2 HDMS mass spectrometer equipped with an ion mobility cell and a nanolockspray source in the positive ion and ‘V’ mode. The doubly-charged ion ([M+2H]^2+^) was used for initial single-point calibration and MS/MS fragment ions of GFP [Glu 1]-Fibrinopeptide B with a [M + 2H]^2+^ = 785,84206 *m/z* was used as lock mass, to obtain the final instrument calibration. Data-independent scanning (MSE) experiments were performed by switching between low (3 eV) and elevated collision energies (15–50 eV), applied to the trap ‘T-wave’ cell filled with argon. Scan time of 0.8 s were used for low and high energy scans from *m/z* 50 to 2000 [34].

### Processing Parameters and Database Search

The raw data processing, protein identification and relative quantitative analyses were all performed using the ProteinLynx Global Server (PLGS- v 2.5.1). The processing parameters included: automatic tolerance for precursor and product ions, minimum of 3 fragment ions matched per peptide, minimum of 7 fragment ions matched per protein, minimum of 2 peptides matched per protein, 1 possible trypsin missed cleavage, carbamidomethylation of cysteine as fixed modification and oxidation of methionine as variable modification, and a maximum false positive discovery rate (FDR) of 4% that was determined based on the search of a reversed database, which was generated automatically using PLGS 2.5.1 by reversing the sequence of each entry.

To identify and quantify the proteins, the intensities of the spectra were calculated by the stoichiometric method, according to the internal standard, the sequence of rabbit phosphorylase (Uniprot entry: P00489), by MSE analysis [34] and normalized using the PLGS auto normalization function. The amount and sequence of the matched peptide and protein *f*mols were obtained, based on the ratio of its three most abundant peptides (“High Top 3” method) determined in each individual experiment [34], considering the data from 3 biological replicates for each sample. All protein hits were identified with a confidence of > 95%. Only those proteins identified in at least two out of 3 replicates performed for each biological sample, were regarded as having undergone a significant change.

Protein identifications were obtained with the embedded ion accounting algorithm of PLGSsoftware searching into the UniProtKB/Swiss (http://www.uniprot.org/ release Version 2015_01) from *Puccinia spp* proteins (28,122 entries) appended in the internal standard. The final expression data was screened according to the following criteria: statistical significant fold change as well as standard deviation (SD) and their correspondent *p* value: *p*≤0.05 and *p*≥ 0.95 were considered whose abundance was statistically increased in the PpEucalyptus and PpGuava populations, respectively. Data labeled as PpEucalyptus and PpGuava represent proteins that were only identified in that population.

After the PLGS 2.5.1 expression analysis the data was manually inspected, and some parameters obtained using the MassPivot v 101.jar were included: (*i*) the average amount (*fmol*) of protein, (*ii*) average score of proteins and (*iii*) average amount of matched peptides to each protein.

### Functional Analysis

The protein sequences that were matched with ionized peptides were functionally categorized by the Gene Ontology Consortium (GO) [35] (http://www.geneontology.org). The terms were obtained using the Blast2GO software with the default parameters specified by the program [36]. These annotations were previously simplified using the GO Slim feature. The EC numbers (Enzyme Commission) were obtained and the prediction of the subcellular localization of the proteins performed using the Signal P (http://www.cbs.dtu.dk/services/SignalP/) and TMHMM (http://www.cbs.dtu.dk/services/TMHMM-2.0/) programs by the InterProScan tool (http://www.ebi.ac.uk/interpro/).

## Results

### Light microscopy analysis and plant infection

**Fig 1:**
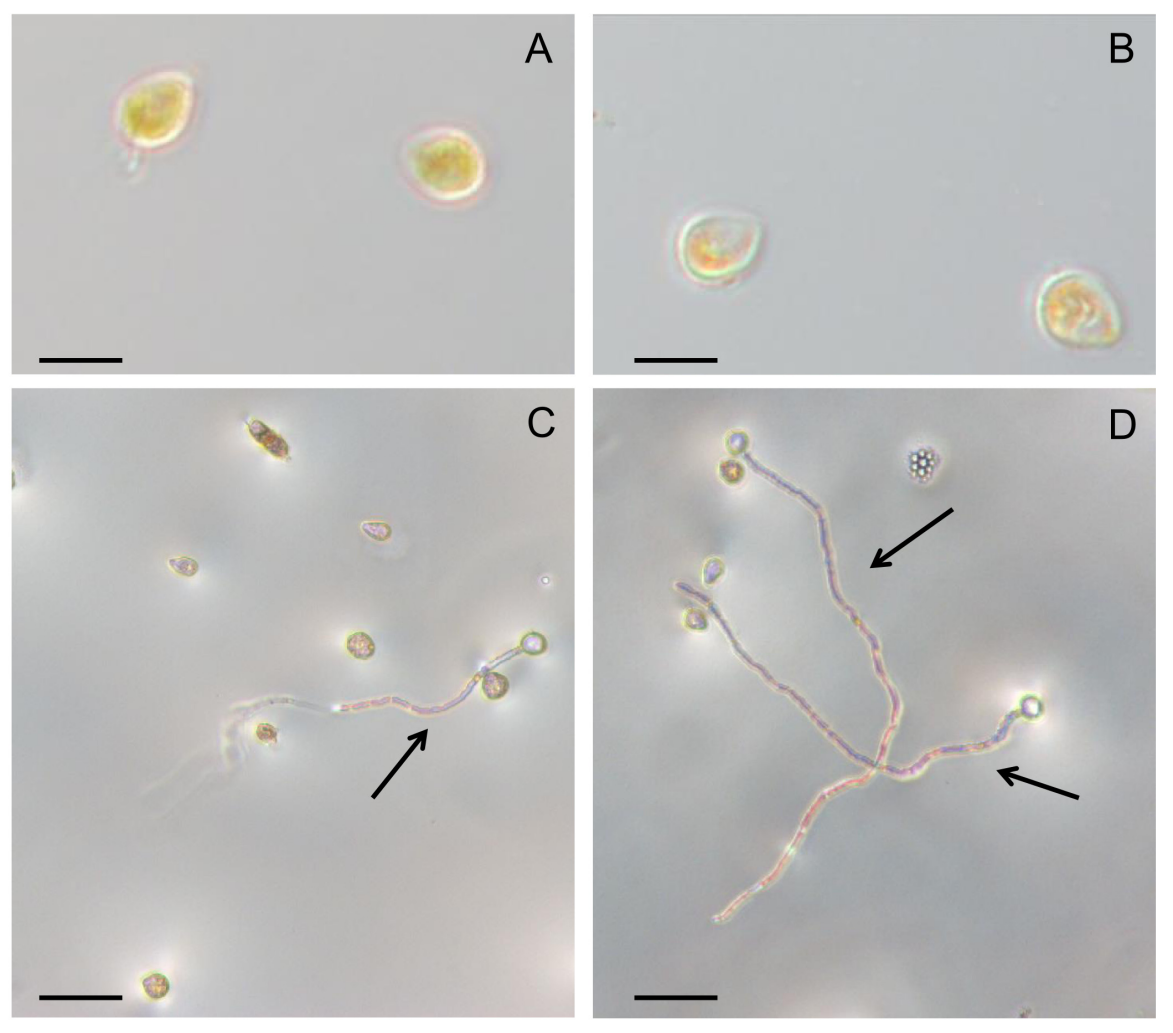
Morphological and viability analysis of *Puccinia psidii* uredospores. *P*. *psidii* uredospores from *E*. *grandis* **(A)** and *P*. *guajava* **(B)** exhibit similar morphology and germination viability, respectively **(C and D)**.The arrows indicate the fungal germ tube in both uredospore populations, 24 hours after inoculation in water-agar medium. Light microscopy images of PpEucalyptus and PpGuava uredospores are shown at 100 X (A and B) and 200 X (C and D) magnification. Scale bar: 20 μm in A and B, 50 μm in C and D.

The morphological analysis showed that the size and shape of the uredospores were similar for both the PpEucalyptus and PpGuava populations (Fig 1A and 1B). The average length of the uredospores were 19.5 ± 2.2 μm and 19.9 ± 0.4 μm, respectively, for the PpEucalyptus and PpGuava populations, and their respective average widths were 13.5 ± 1.9 μm and 11.7 ± 0.7 μm, suggesting that there was no relationship between the uredospore size or shape and virulence or physiological variability of the populations. The populations also showed similar uredospore viability under *in vitro* conditions, with approximately 25% of the spores germinating on water-agar media (Fig 1C and 1D).

**Fig 2:**
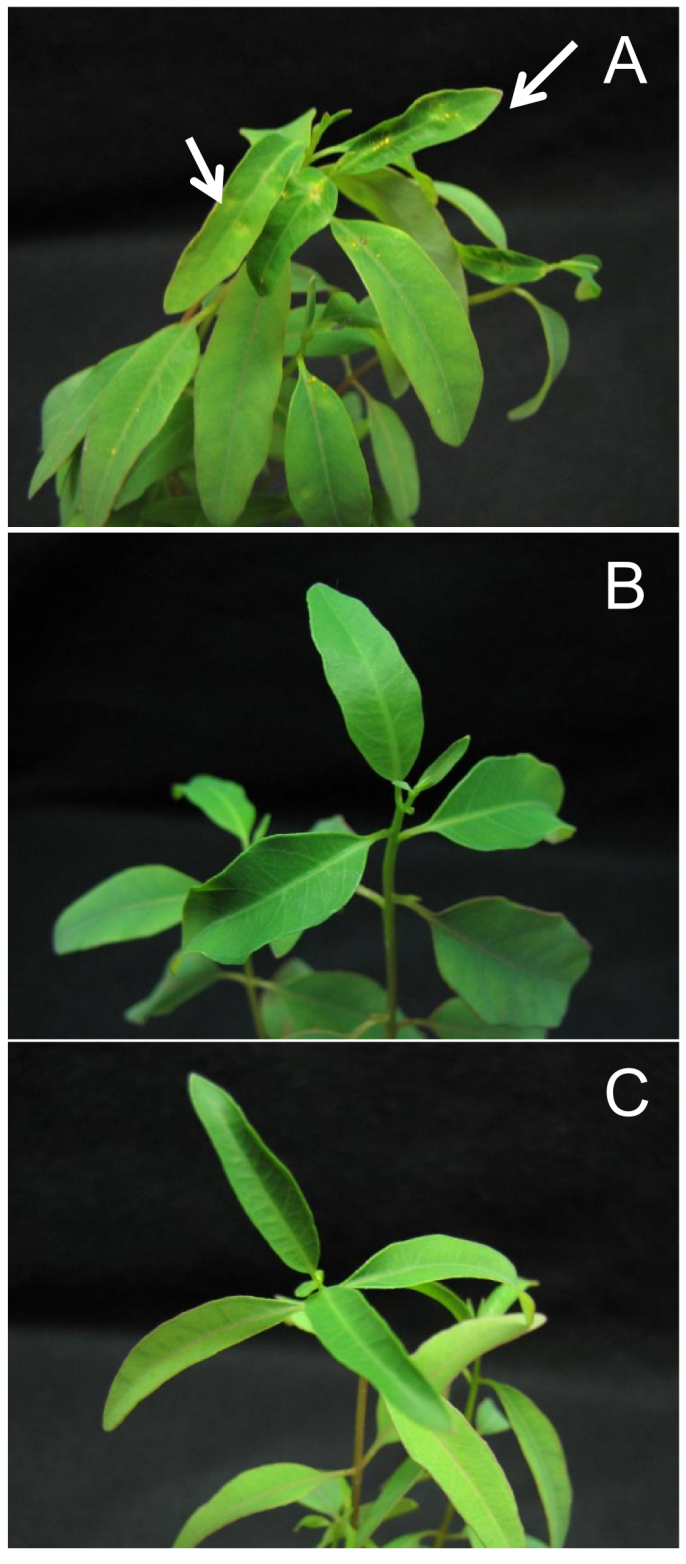
Eucalyptus infection by guava and eucalyptus rust. Symptoms induced by inoculation of *P*. *psidii* uredospores from PpEucalyptus **(A)** on *E*. *grandis* variety D901. This clone is rust susceptible when grown under field conditions. The white arrows indicate the fungal pustules. The leaves are shown 15 days after inoculation.The PpGuava populations **(B)** and control **(C)** did not show typical rust symptoms.

Two weeks after inoculating *E*. *grandis* plantlets, uredospore pustules were visible on the host leaves inoculated with PpEucalyptus (Fig 2A), whereas the plantlets inoculated with PpGuava (Fig 2B) and the control plantlets (Fig 2C) did not exhibit any rust symptoms. The absence of pustules, chlorotic flecks or leaf deformation, demonstrate the existence of physiological variability and pathogenic specificity between the sampled populations.

### Overview of the *P*. *psidii* uredospore proteome

**Fig 3:**
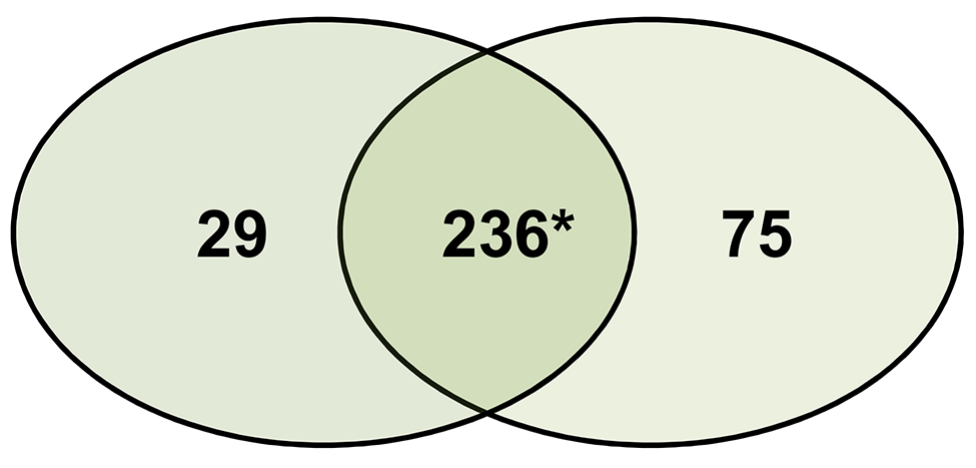
Proteins identified from *P*. *psidii* uredospore populations. The proteins exclusively found in PpGuava (right) and PpEucalyptus (left) and common to both populations (center). Of the common proteins, 25 and 120 whose abundance were increased in PpEucalyptus and PpGuava, respectively.

In total, 340 proteins were identified using the UniProt *Puccinia* genera sequences (UniProt database). Using spectral counting based label free quantification, we observed changes in relative protein abundance and a greater amount of proteins up regulated in PpGuava (S1 Table). Seventy five proteins were found exclusively in PpGuava and 29 in PpEucalyptus. Among the common proteins, 25 and 120 were increased in PpEucalyptus and PpGuava respectively (Fig 3).

Table 1 shows some significant differences between PpGuava and PpEucalyptus protein profiles. Proteins commonly found in fungal proteome studies were identified in both fungal populations, such as ribosomal subunits, elongation factors and tubulins, as well as proteins related to energy generation, such as ATP synthase subunits.

**Table 1:** Identification of proteins that were differentially represented in the *P*. *psidii* uredospore populations from *E*. *grandis* (PpEucalyptus) and *Psidium guajava* (PpGuava).

Accession^a^	Description	Score^b^	Ratio ^c^	Log Ratio ^d^	SD^e^	p value^f^
E3L509	1 4 alpha glucan branching enzyme	480.40	1.28	0.25	0.23	0.98
E3L9C1	14 3 3 family protein	12200.67	1.42	0.35	0.12	1.00
E3L0L2	2 isopropylmalate synthase	147.74	1.20	0.18	0.10	1.00
E3KG66	2 methylcitrate dehydratase	399.00	1.42	0.35	0.25	0.99
E3KHM1	26S protease regulatory subunit 8	2686.01	1.12	0.11	0.14	0.95
E3K1H4	26S protease subunit rpt4	2005.47	1.27	0.24	0.16	0.98
E3K877	26S proteasome non-ATPase regulatory subunit 14	1971.74	1.51	0.41	0.16	1.00
E3KKA1	26S proteasome regulatory subunit 6A	2758.75	1.75	0.56	0.13	1.00
E3KZT9	26S proteasome regulatory subunit 7	708.37	1.22	0.20	0.19	0.96
E3JZ59	3 isopropylmalate dehydratase	483.41	1.25	0.22	0.16	1.00
E3L5E7	40S ribosomal protein S1	801.36	PpGuava	PpGuava	PpGuava	PpGuava
E3JWG6	40S ribosomal protein S11	2195.29	0.31	-1.16	0.24	0.00
E3KBT0	40S ribosomal protein S16	1362.80	1.70	0.53	0.23	1.00
E3K5L6	40S ribosomal protein S19 A	7449.16	0.64	-0.44	0.16	0.00
E3JVN0	40S ribosomal protein S21	4844.32	1.62	0.48	0.16	1.00
E3L8H2	40S ribosomal protein S3	675.32	1.60	0.47	0.21	1.00
E3KJL1	40S ribosomal protein S5	506.24	0.66	-0.42	0.27	0.00
E3K826	60S acidic ribosomal protein L10	1395.34	2.41	0.88	0.16	1.00
E3KKV5	60S ribosomal protein L14	1806.10	0.20	-1.59	0.44	0.00
E3KGR6	60S ribosomal protein L23	3152.83	PpGuava	PpGuava	PpGuava	PpGuava
E3KZR5	60S ribosomal protein L9e	687.84	1.37	0.32	0.29	0.98
E3KVF5	Acetyl CoA carboxylase biotin carboxylase	465,35	PpEucalyptus	PpEucalyptus	PpEucalyptus	PpEucalyptus
E3L3M4	Acetyl CoA acetyltransferase	5467.85	1.71	0.54	0.13	1.00
E3KLM7	Acetyl-CoA acyltransferase	2220,71	1.62	0.48	0.15	1.00
E3KL13	Acetylglutamate kinase	256.81	1.14	0.37	0.19	0.99
E3K4I1	Actin	25906.00	1.16	0.15	0.05	1.00
E3JQQ0	Acyl coenzymeA oxidase	384.27	1.46	0.38	0.15	1.00
E3JUV4	Acyl-CoA dehydrogenase	259.56	1.80	0.59	0.22	1.00
E3JPY7	ADP ribosylation factor	1302.03	1.39	0.33	0.23	1.00
E3KWD3	ADP.ATP carrier protein	6064.67	1.27	0.24	0.09	1.00
E3KIY1	AGC/AKT protein kinase	392.41	1.36	0.31	0.24	0.99
E3JT53	Argininosuccinate synthase	2357.95	1.30	0.26	0.15	1.00
E3L109	ASF1 like histone chaperone	521.43	PpEucalyptus	PpEucalyptus	PpEucalyptus	PpEucalyptus
E3KQV7	Aspartate aminotransferase *	3436.00	1.45	0.37	0.11	1.00
E3KQV8	Aspartate aminotransferase *	1152.40	1.62	0.48	0.18	1.00
E3K898	Aspartate tRNA ligase	397.95	1.31	0.27	0.21	0.99
E3KZB7	ATP synthase subunit alpha	15253.20	1.32	0.28	0.05	1.00
E3K357	ATP synthase subunit beta	24197.30	1.28	0.25	0.06	1.00
E3K775	Autocrine motility factor receptor	878.26	2.01	0.70	0.21	1.00
E3KLJ3	Calmodulin	2518.71	1.45	0.37	0.19	0.99
E3JYU7	Capping protein (Actin filament) muscle Z-line. beta	484.72	PpGuava	PpGuava	PpGuava	PpGuava
E3KXV8	Catalase	1294.27	1.22	0.20	0.12	1.00
E3KRP0	cell division cycle protein 48	10137.00	1.25	0.22	0.06	1.00
E3L2Y3	Citrate synthase *	3648.19	1.45	0.37	0.07	1.00
E3KIG5	Citrate synthase *	287.31	PpGuava	PpGuava	PpGuava	PpGuava
E3KQN1	CK1/CK1/CK1-D protein kinase	356.34	PpGuava	PpGuava	PpGuava	PpGuava
E3K4L0	Coronin	335.26	1.28	0.25	0.17	1.00
E3JW29	Cytochrome c	1794.11	1.17	0.16	0.12	1.00
E3L8N2	Cytochrome c oxidase subunit Va	939.33	1.79	0.58	0.46	0.98
E3KXK0	Enolase	13598.00	1.36	0.31	0.08	1.00
E3JRG6	Epsin 3	210.54	PpEucalyptus	PpEucalyptus	PpEucalyptus	PpEucalyptus
E3JUT0	Fructose-bisphosphate aldolase. class II	2168.36	0.68	-0.38	0.19	0.00
E3K026	GDP mannose 4 6 dehydratase	230.21	1.48	0.39	0.22	1.00
E3KEB5	Glucose 1-dehydrogenase	363.08	PpEucalyptus	PpEucalyptus	PpEucalyptus	PpEucalyptus
E3K5I2	Glucose 6 phosphate 1 dehydrogenase	4756.44	1.25	0.22	0.21	0.99
E3L278	Glutathione reductase (NADPH)	679.20	1.27	0.24	0.21	1.00
E3L363	Glycerol 3 phosphate dehydrogenase	1383.98	1.43	0.36	0.12	1.00
E3KA23	Glycyl-tRNA synthetase	443.03	1.16	0.15	0.14	0.99
E3K2A8	GTP-binding nuclear protein spi1	3836.23	1.35	0.30	0.09	1.00
E3KXR7	Guanine nucleotide-binding protein subunit beta-like protein	9323.70	1.36	0.31	0.11	1.00
E3K5H8	Heat shock 70kDa protein 4	1721.81	1.75	0.56	0.09	1.00
E3KZR1	Heat shock protein 60	7677.27	1.54	0.43	0.06	1.00
E3JVS0	Heat shock protein 83	16505.40	1.17	0.16	0.04	1.00
E3KYT3	Heat shock protein HSS1 *	15718.00	1.16	0.15	0.05	1.00
Q01877	Heat shock protein HSS1 *	11951.00	PpGuava	PpGuava	PpGuava	PpGuava
E3K643	Heat shock protein SSB	12675.20	1.27	0.24	0.06	1.00
E3K1Q1	Heme-binding peroxidase	6757.64	1.73	0.55	0.14	1.00
E3L1S2	Histone H2A	7513.68	0.92	-0.08	0.08	0.03
E3KEI6	Histone H2B	6731.25	0.63	-0.47	0.14	0.00
E3JQ71	Histone-binding protein RBBP4	790.83	PpGuava	PpGuava	PpGuava	PpGuava
E3KZR0	Hsp10 like protein	1463.97	1.45	0.37	0.15	1.00
E3JT24	Hsp70-like protein	2010.73	1.45	0.37	0.10	1.00
E3JX32	Hydroxymethylglutaryl CoA synthase	271.48	1.42	0.35	0.20	1.00
E3KHG9	Inorganic pyrophosphatase	8350.30	1.22	0.20	0.09	1.00
E3K023	Isocitrate lyase	821.33	1.19	0.17	0.10	1.00
E3K387	Ketol-acid reductoisomerase, mitochondria	3191.20	1.57	0.45	0.11	1.00
E3L7N3	Long chain fatty acid CoA ligase 1	269.38	PpEucalyptus	PpEucalyptus	PpEucalyptus	PpEucalyptus
E3K352	Malate dehydrogenase *	2264.81	1.82	0.60	0.17	1.00
E3L321	Malate dehydrogenase *	4555.28	1.82	0.60	0.10	1.00
E3KNM2	Mannose 1 phosphate guanyltransferase	2499.70	1.39	0.33	0.13	1.00
E3JQQ5	Minichromosome maintenance protein 3	315.62	1.43	0.36	0.16	1.00
E3JQ11	Mitochondrial processing peptidase subunit beta	597.65	1.55	0.44	0.17	1.00
E3KGF0	Myo-inositol-1-phosphate synthase	290.58	1.43	0.36	0.27	0.99
E3K8H6	NADH dehydrogenase (Ubiquinone) Fe-S protein 3	272.99	1.68	0.52	0.33	1.00
E3LB27	NADH dehydrogenase flavoprotein 2	1752.80	3.49	1.25	0.39	1.00
E3LBN8	Peptidyl-prolyl cis-trans isomerase	416.71	PpEucalyptus	PpEucalyptus	PpEucalyptus	PpEucalyptus
E3JVD3	Phospho 2 dehydro 3 deoxyheptonate aldolase	553.04	PpGuava	PpGuava	PpGuava	PpGuava
E3L907	Phosphoenolpyruvate carboxykinase	5909.17	1.73	0.55	0.11	1.00
E3K5I5	Phosphoglucomutase	1874.05	1.40	0.34	0.12	1.00
E3KDN8	Phosphomannomutase	2918.24	1.63	0.49	0.13	1.00
E3KGF8	Prohibitin 1	1398.25	1.84	0.61	0.16	1.00
E3K554	Proteasome subunit alpha type	2947.92	1.43	0.36	0.20	1.00
E3KIE7	Proteasome subunit beta type	386.94	PpEucalyptus	PpEucalyptus	PpEucalyptus	PpEucalyptus
E3JXB4	Protein transporter SEC23	218.15	1.73	0.55	0.29	1.00
E3KY42	Pyridoxine biosynthesis protein	3497.74	1.22	0.20	0.12	1.00
E3KTX9	Pyruvate carboxylase	1507.30	1.12	0.11	0.07	1.00
E3KSI6	Pyruvate dehydrogenase E1 component subunit alpha	211.05	1.43	0.36	0.17	1.00
E3KF45	Rab family protein	413.58	1.70	0.53	0.32	1.00
E3KHK5	Ribose 5 phosphate isomerase	205.02	1.54	0.43	0.18	1.00
E3KNA3	S25 ribosomal protein	1314.31	PpEucalyptus	PpEucalyptus	PpEucalyptus	PpEucalyptus
E3KNU8	S-adenosylmethionine synthase	1648.47	1.67	0.51	0.17	1.00
E3JXY4	Serine/threonine-protein phosphatase PP1	187.58	1.63	0.49	0.41	1.00
E3JS02	Small COPII coat GTPase	1604.51	1.57	0.45	0.25	1.00
E3KYA0	Small nuclear ribonucleoprotein D3	852.35	PpEucalyptus	PpEucalyptus	PpEucalyptus	PpEucalyptus
E3JQK5	Small nuclear ribonucleoprotein E	2077.40	PpGuava	PpGuava	PpGuava	PpGuava
E3K8I4	Stress induced phosphoprotein 1	532.99	1.36	0.31	0.26	0.99
E3KK64	Succinate dehydrogenase flavoprotein subunit mitochondrial	2231.15	1.49	0.40	0.14	1.00
E3KMN8	Thioredoxin reductase	529.23	1.79	0.58	0.13	1.00
E3JSQ1	Transaldolase	3002.86	1.30	0.26	0.13	1.00
E3JPZ9	Triosephosphate isomerase	7959.44	1.32	0.28	0.09	1.00
E3KK52	Tryptophan synthase	218.58	1.27	0.24	0.13	1.00
E3JT05	Tubulin alpha-1A chain	5271.32	1.16	0.15	0.10	0.99
E3KPL3	Tubulin beta chain	18116.00	1.45	0.37	0.04	1.00
E3K479	tubulin binding cofactor A	689.77	1.51	0.41	0.21	1.00
E3KA35	U6 snRNA-associated Sm-like protein LSm3	1905.08	1.36	0.31	0.33	0.97
E3KBL0	Ubiquitin-activating enzyme E1	515.66	1.26	0.23	0.14	1.00
E3KLK1	Ubiquitin-conjugating enzyme E2 N	1681.96	1.52	0.42	0.21	1.00
E3K7I4	UDP-glucose 4-epimerase	358.18	0.77	-0.26	0.22	0.03
E3K0X4	UDP-glucose 6-dehydrogenase	3766.77	1.19	0.17	0.08	1.00
E3L4X0	UTP-glucose-1-phosphate uridylyltransferase	2906.12	1.32	0.28	0.07	1.00
E3L015	Vacuolar type proton ATPase catalytic subunit A	6515.19	1.20	0.18	0.08	1,00

^a^ Access ID is the access identification of the protein in the Uniprot database (http://www.uniprot.org/ release Version 2014_08).

^b^ The score of protein expression analysis

^c and d^ Ratio and the Log (PpGuava/PpEucalyptus) of quantified proteins respectively

^e^ Standard Deviation (SD) of the Log of PpGuava/PpEucalyptus ratio and ^f^p-value of Log of PpGuava/PpEucalyptus ratio obtained from PLGS 2.5.1.

A *p value* ≤0.05 identifies proteins less abundant, whereas a *p value* ≥0.95 identifies proteins more abundant in PpGuava than in PpEucalyptus; *p value* identifies proteins that were unique to either the PpEucalyptus or PpGuava *P*. *psidii* uredospore populations isolated from eucalyptus and guava, respectively.

* Variant proteins.

The functional analysis showed a clear predominance of proteins related to stress response, such as heat shock proteins, catalases that were more abundant in PpGuava than in PpEucalyptus. Proteins related to fungal pathogenesis such as, citrate synthase and enolase, among other, were also unique or increased in PpGuava population. Additionally, fatty acid synthase was unique in the PpEucalyptus population.

Some proteins were found to have one or more variants, with different abundance levels. Our results suggest that there are a variety of protein homologs in the uredospores; for example, there were matches for 4 different malate dehydrogenase proteins and 2 matches for pyruvate carboxylase. Moreover, 167 proteins were described as uncharacterized proteins with different degrees of abundance (S1 Table).

### Functional categorization of identified proteins

**Fig 4:**
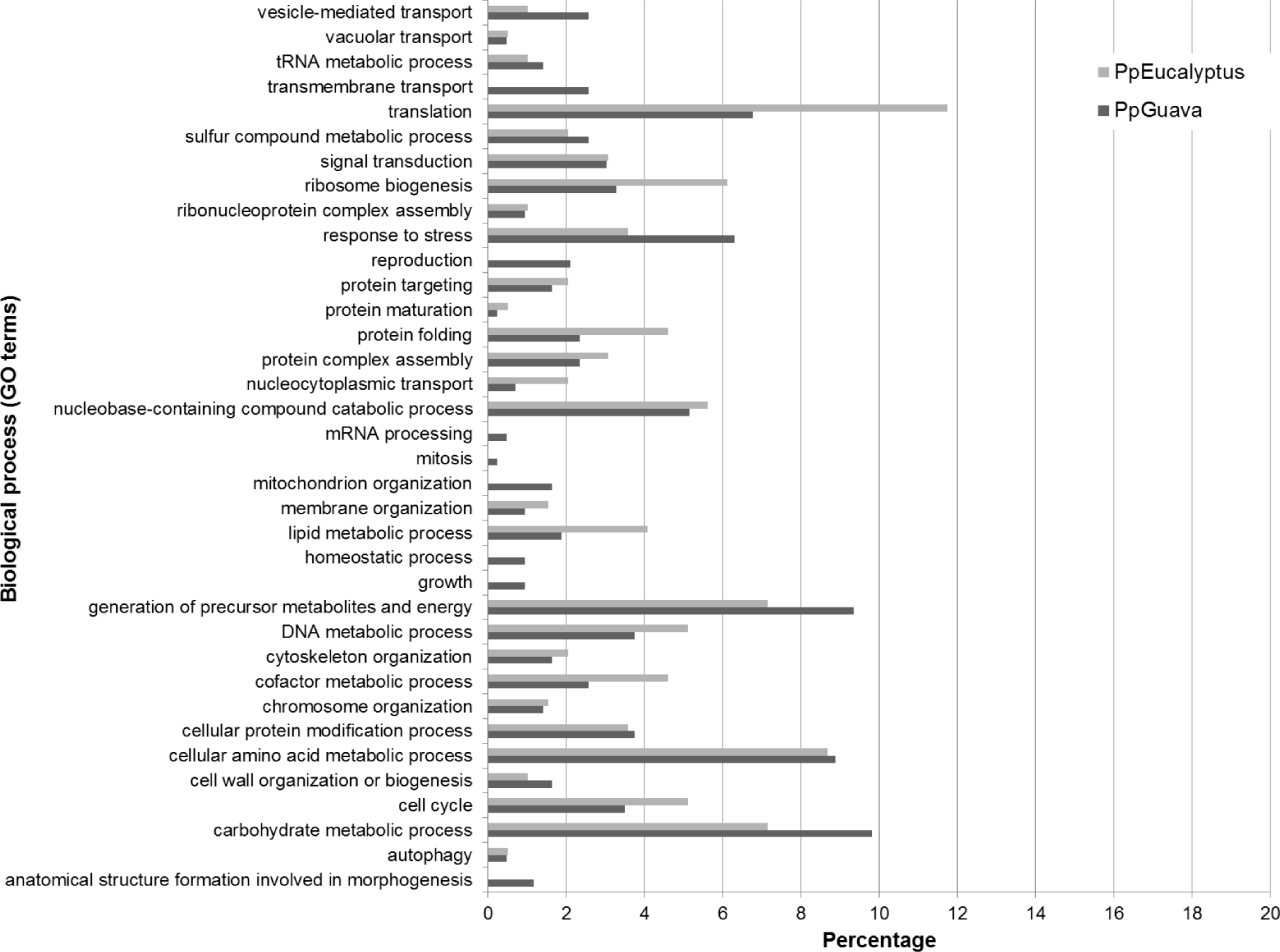
Gene ontology of biological process terms in the proteomic analysis. Bar graph represents the ratio of % composition of term in the proteomic data.

Proteins were categorized according to biological process using the Blast2GO software and major categories were present in both populations, such as cellular amino acid metabolic process, carbohydrate metabolism process, generation of precursor metabolites and energy, cellular protein modification process, signal transduction, vacuolar transport and others (Fig 4). However some GO terms, such as anatomical structure formation involved in morphogenesis, growth, mitosis, reproduction, transmembrane transport, mRNA processing, homeostatic process, mitochondrion organization, were only represented by the unique or more abundant from the PpGuava than PpEucalyptus population.

This information and more details about the proteins as the Enzyme Code, Signal P and the presence of transmembrane helice domain (TMHMM), as well as proteins scores and peptide sequence are listed in S2 Table.

## Discussion

*P*. *psidii* s.l. is unusual rust in that it exhibits a wide host range and is known to infect over 33 plant genera and 129 species, primarily in the Myrtaceae [37, 38, 39]. Formerly, *P*. *psidii* s.l. is conspecific and has been known as guava or eucalyptus rust [4, 40, 41, 42] due to the importance of these two hosts to the pathogen.

Despite the rust symptoms in the field being more pronounced in guava, cross-infection using PpGuava uredospores did not result in visible rust symptoms on *E*. *grandis*. However, light microscopy analysis of the PpEucalyptus and PpGuava uredospores showed the ability of both to produce germ tubes under *in vitro* conditions and probably having the potential to infect the host. Differences in the pathogenesis of *P*. *psidii* population’s were first reported by Maclachlan [43]. Since then, several cross-infection studies involving several hosts and *P*.*psidii*, collected from different plants of the Myrtle family, have shown clear differences in the pathogenicity of the organism [4, 13, 14, 44, 45, 46]. Aparecido et al. [14] inoculated five different hosts (*Corymbia citriodora*, *P*. *guajava*, *S*. *jambos*, *Eugenia sp*., and *Eugenia uvalha*) with uredospores collected from eight different host species.From this experiment, the authors described four distinct pathogenic groups. In our study, the PpGuava did not infected *E*. *grandis*, or there were no visible symptoms. It suggests the existence of a host-specific interaction, as a natural physiological variability among the uredospore populations. The differences observed in their protein profiles may be explained by the natural genetic variability within the investigated fungal populations. Our data is corroborated by recent molecular analysis of *P*. *psidii* populations that clearly proved that there is a correlation between the genetic variability of the *P*. *psidii* populations and the host from which they were collected [6].

Although rust is an economically important disease in eucalyptus and in other myrtle cultures, very little is known about the changes in the proteome of different populations of *P*. *psidii* s.l. Moreover, the monitoring of the changes in the uredospores protein profile, according to original host, is a new perspective to better understanding the plant-microbe interactions.

We evaluated two populations of the same fungal species, and observed a large number of proteins whose levels changed between the samples, as well as the presence of unique proteins in both samples. We opted to perform fungal population protein analysis based on the fact that the *P*. *psidii* has a biotrophic life style. There is no report of its growth and multiplication on synthetic media [6]. Being unable to grow outside of the host, it is difficult to characterize the proteome from other fungal structure. Therefore, it is laborious to produce spores using susceptible plants for growth and purification of the isolates. During this process, artificial selection may occur potentially producing a more aggressive pathogen depending on the host.

Some studies, which used different methods and equipments, found larger number of proteins than the 340 described for *P*. *psidii*. For instance, Bindschedler et al. [47] found 441 proteins from ungerminated spore of *Blumeria graminis* f. sp. *Hordei*. Cooper et al. [48] classified approximately 400 proteins from asexual uredospores of *Uromyces appendiculatus* and Liang et al. [49] observed the presence of 719 proteins from the three stages of sclerotia formation of *Sclerotinia sclerotiorum*. The lack of a *P*. *psidii* database was a strong challenge in the proteomic studies. The protein spectrums were matched with those from *Puccinia* genus from Uniprot. *P*. *psidii* has a non-complete genome sequenced. There are just few proteins sequences from this organism [50]. Many of these proteins are beta tubulins and transcription factors, which points a difficulty for protein identification of *P*. *psidii* uredospores. Even though, apart from the difficulties, we could have a good view of the protein roles in the uredospores between two contrasting *P*. *psidii* populations.

Using uredospores from populations that have been previously described as different biotypes [13], we identified proteins that are potentially related to the degree of variability and aggressiveness of the *P*. *psidii* allowing us to make some inferences regarding fungal diversity. A larger number of unique and/or abundant proteins were found in the PpGuava uredospores; this is most likely to be related to the higher genetic diversity within PpGuava, resulting in a broader range of proteins produced by this population, as well as a larger number of possible isoforms, which may be justified by the probable higher number of alleles in this population. These data are in agreement with a recent study that compared rust populations from eucalyptus and other myrtle species in Brazil and revealed a higher genetic diversity in the guava populations [6].The authors suggested that the pathogen recently migrated from a native South American host. When the fungus moved to other plant species, such as the exotic *Eucalyptus* spp., a new selection process was introduced by the new host [6]. Graça et al. [39] did not prove that *P*. *psidii* jumped from guava to eucalyptus host, but the existence of host-associated biotypes of *P*. *psidii* in Brazil, indicates that this diversity must be considered. Morin et al. [51] investigated the host-range of the *P*. *psidii* s.l. present in Australia. The authors concluded that the development of the disease in one host species in a tribe did not mean that a related species or genus also developed the disease. Moreover, they demonstrated the inability of the Australian rust accession to develop uredinia on *P*. *guajava* compared to the accession from Florida [52], supporting the hypothesis of physiological diversity between these populations.

Interestingly, during the uredospore phase, we identified many proteins related to fungal pathogenicity, such as peptidases, proteases, and proteins that are able to modify host factors [53, 54, 55, 56, 57], that were unique or more abundant only in the PpGuava than in PpEucalyptus population, for instance, proteassomes. Proteasomes are multi-subunit and multicatalytic proteolytic complexes that are found in the cells of most organisms, and these complexes play important roles in protein turnover in both the cytosol and the nucleus. They selectively degrade intracellular proteins in eukaryotic cells, particularly *via* the ubiquitin-proteasome pathway [58], and are involved in multiple specific functions, such as cell cycle regulation, cell signaling, the selective elimination of abnormal proteins, and the flux of substrates through metabolic pathways [59, 60]. As previously suggested by Kim et al.[61], the wax coated hydrophobic surfaces, which naturally occur on the host surface, may activate the 20S proteasome to mobilize the storage proteins that are present in uredospores. Chaperones are responsible for the initial folding and maintenance of proteins structure compared to the conventional heat shock proteins (HSPs), which are present only under conditions of high temperature or stress. Some studies suggested that these proteins also act to prevent protein aggregation, thus controlling protein turnover, which will increase its longevity and virulence [62]. Hodgetts et al. [63] observed that the up-regulation of HSP90 in *Saccharomyces cerevisiae* resulted in an increase of virulence in infected mice. We identified many HSP classes, some with peptide signals for secretion, similar to the data reported by Song et al [30]. The authors found high level of HSP in *P*. *triticina* haustorium. All identified HSPs were less abundant in the PpEucalyptus than in PpGuava population, most likely because the uredospores from the guava fruit are under more stressfull conditions [47] than those on PpEucalyptus, suggesting a differential response to the host organism. Larger amounts of HSPs were also identified in the *Uromyces appendiculatus* uredospores, which cause bean rust [48]. More investigation into the expression of HSPs is needed to better understand the roles of these proteins in the infection process during the *P*. *psidii* s.l. life cycle.

Tubulin and actin proteins were also increased in abundance in PpGuava. Similarly, Mandelc et al. [32] also observed that these proteins were increased in abundance in the lethal phytotype of *V*. *albo-atrum*. Tubulins and actins are the major proteins involved in the formation of the microtubules and microfilaments. GTPases regulates cellular polarization and motility, which affects the cytoskeleton, and are involved in the regulation of the secretion process and endocytosis [64]. The enolases identified in this study were also higher in abundance in the PpGuava population. Enolases participate in other cellular activities and can even act as HSPs; they can bind to cytoskeletal and chromatin structures to modulate transcription, thus playing a crucial role in pathophysiological processes [65, 66]. Enolase proteins increased it abundance in a virulent isolate of *Pyrenophora tritici-repentis*, the causal agent of tan spot in wheat [67]. Another example is the increased abundance of malate dehydrogenase in PpGuava. This protein is related to fungal virulence, the secretion of this compound acidifies the environment, signaling the expression and secretion of virulence factors and phytotoxins [17, 32].

Another interesting protein class, not previously related to fungal virulence, is prohibitin. This protein, with homologues in animals, fungi, plants, and unicellular eukaryotes was first reported as a negative regulator of cell proliferation. However, prohibitin is expressed as two transcripts with varying lengths of the 3' untranslated region. The longer one is present at higher levels in proliferating cells, suggesting that this longer 3' untranslated region sequence may function as a trans-acting regulatory RNA [68, 69].

Acetyl CoA carboxylase biotin carboxylase is abiotin-dependent enzyme involved in the biosynthesis, as well as, in the metabolism of fatty acids [70, 71]. Fatty acid synthase catalyzes the formation of long-chain fatty acids from acetyl-CoA, malonyl-CoA and NADPH [72]. Both were found as PpEucalyptus unique proteins., maybe in PpGuava these proteins may be present in lower abundance.

Our data also support the model proposed for *U*. *appendiculatus* by Cooper et al. [48], which suggests that the uredospore exists in a suspended translational state. Uredospores can produce proteins very quickly without having to assemble *de novo* translational components. Moreover, the peptide chains dispersed in the ribosome are protected by heat shock proteins and can resist adverse conformational changes caused by desiccation or high temperatures that the uredospores might be subjected to, once released into the environment [48].Corroborating this hypothesis, we identified a large number of ribosomal proteins subunits with different level of abundance, which may be related to the beginning of the infection process by the pathogen.

Additionally, categorization by protein function showed increased abundance of proteins related to translation, ribosome biogenesis and protein folding from the PpEucalyptus sample. Response to stress, generation of precursor metabolites and energy and carbohydrate metabolic process were more abundant in PpGuava. The proteins involved in signal transduction were present in similar proportion in both populations.

**Fig 5:**
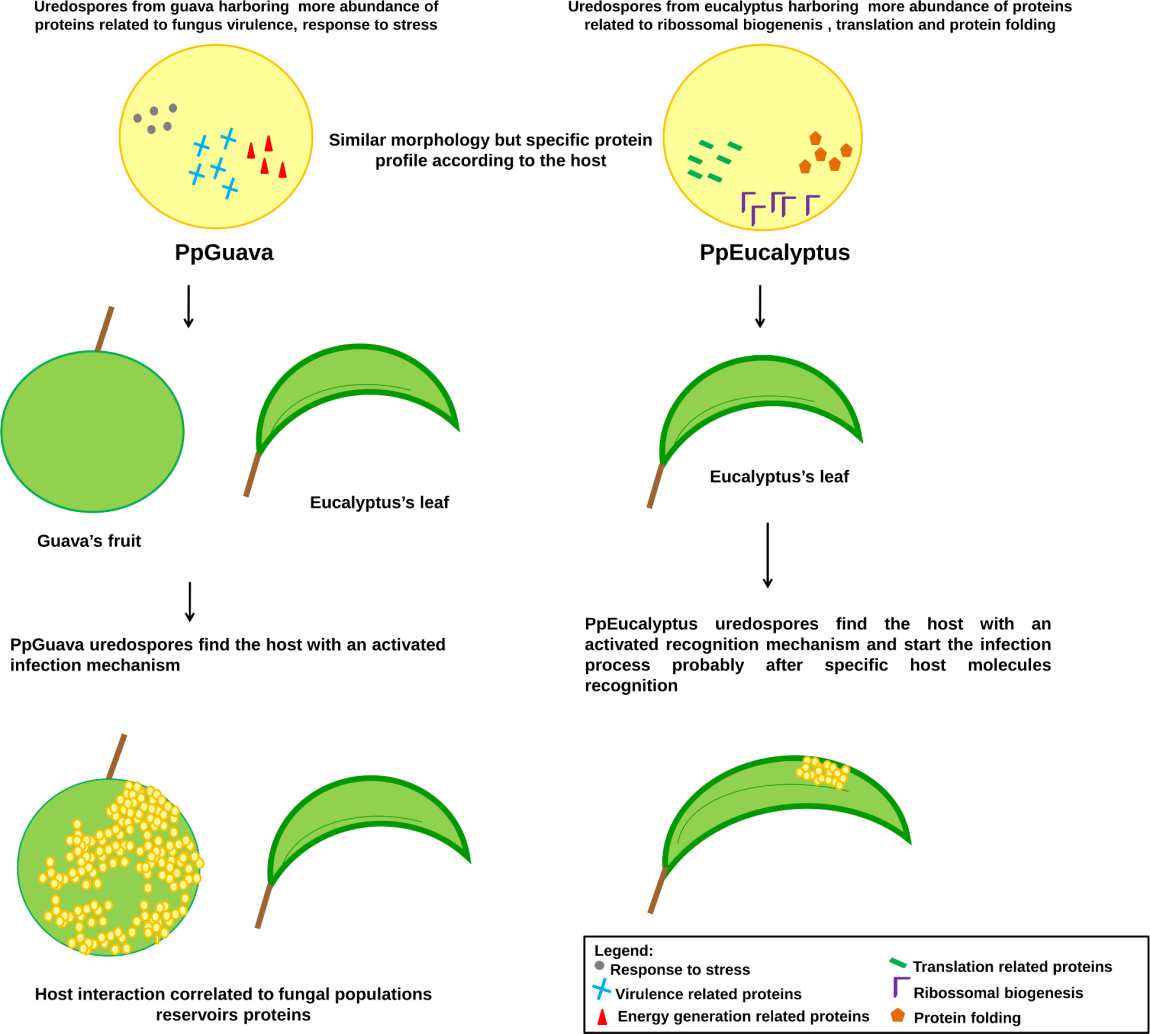
Protein profiles in PpGuava and PpEucalyptus and the correlation with their physiological variability. While proteins correlated to fungal virulence and stress response had the abundance increased in PpGuava, proteins related to biogenesis, protein folding and translation had the abundance increased in PpEucalyptus.

In turn, uredospores populations have specific reservatory of proteins according to the host from supporting the physiological variability of these two populations. The virulent proteins from PpEucalyptus were less abundant when compared to PpGuava. In guava, close to 100% of the fruits can be lost due to severe infections most likely because the uredospores reservatory of proteins of this population; related with virulence and stress response. Likely, PpEucalyptus uredospores need to process of proteins of virulence to start the host infection process, its reservatory is composed by biogenesis, protein folding and translation (Fig 5). It is commonly observed that uredopores from a specific host are less aggressive when infecting a host from other species. The aggressiveness increases after successive cycles of infection in the new host. One hypothesis is the modulation of the expression of some genes according to the host, resulting in different protein reservoirs in each population.

The proteomic information generated in the present work contributes to a better understanding of the mechanisms underlying the pathogenicity of *P*. *psidii* s.l. and its interaction with its host organism. More comprehensive proteomic analyses of the *P*. *psidii*-host interaction, combined with other studies, will be important for elucidating the molecular basis of the interactions in this intriguing pathosystem.

## Supporting Information

S1 Table Complete set of proteins identified using the PLGS 2.5.1 expression analysis. Access ID is the access identification of the protein in Uniprot database (http://www.uniprot.org/ release Version 2015_02). The score of protein expression analysis, ratio and Log (PpGuava/PpEucalyptus) of quantified proteins, standard deviation (SD) of the PpGuava/PpEucalyptus ratio log and their p-values of the Log of the PpGuava/PpEucalyptus ratio obtained from PLGS 2.5.1. A *p value* ≤ 0.05 identifies proteins whose abundance decrease, whereas a *p value* ≥ 0.95 identifies proteins whose abundance increased in PpGuava; *p value* identifies proteins that were unique to either the PpEucalyptus or PpGuava *P*. *psidii* uredospore populations isolated from eucalyptus and guava, respectively (XLS).
(XLSX)

S2 Table Additional information of set of proteins. Organism source, GO biological process, Enzyme Code, Signal P, Trans-membrane helice domain (TMHMM), as well as protein score, average amount (*fmol*) of protein, average score of proteins and peptide sequences and average amount of matched peptides to each protein.
(XLSX)
